# Aspirin modulates inflammatory biomarkers in patients with subcortical silent brain infarcts

**DOI:** 10.3389/fnagi.2024.1507683

**Published:** 2025-01-07

**Authors:** Wonjae Sung, Young Seo Kim, Kyu-Yong Lee, Jae-A Jung, Hojin Choi, Young Joo Lee, Seong-Ho Koh

**Affiliations:** ^1^Department of Neurology, College of Medicine, Hanyang University Seoul Hospital, Seoul, Republic of Korea; ^2^Department of Neurology, College of Medicine, Hanyang University Guri Hospital, Guri, Republic of Korea; ^3^Department of Plastic and Reconstructive Surgery, Hanyang University Guri Hospital, Hanyang University College of Medicine, Guri, Republic of Korea; ^4^Department of Translational Medicine, Graduate School of Biomedical Science & Engineering, Hanyang University, Seoul, Republic of Korea

**Keywords:** subcortical silent brain infarct, aspirin, inflammation, macrophage migration inhibitory factor, matrix metalloproteinase-9, visfatin

## Abstract

**Introduction:**

This study aimed to identify differences in the levels of inflammation-related biomarkers between patients with subcortical silent brain infarcts (SBIs) and healthy controls. We also evaluated the effect of aspirin on the subcortical SBI inflammatory processes.

**Methods:**

Consecutive patients diagnosed with subcortical SBIs without a history of acute stroke were included. The demographic and clinical data of the 26 subjects with subcortical SBIs, such as the number and location of subcortical SBIs, were reviewed. Plasma levels of macrophage migration inhibitory factor (MIF), matrix metalloproteinase-9 (MMP-9), and visfatin were measured in patients with subcortical SBIs and ten healthy participants. These biomarkers were rechecked in patients with subcortical SBI 3 months after taking aspirin (100 mg/day).

**Results:**

MIF and MMP-9 levels were significantly higher in patients with subcortical SBIs than in healthy control group (*p* = 0.031 and *p* = 0.026, respectively). Although MIF and MMP-9 did not show significant changes after taking aspirin for 3 months, the median plasma level of visfatin was significantly decreased from 1.00 ng/mL (range, 0.86–1.16 ng/mL) to 0.84 ng/mL (range, 0.77–0.91 ng/mL) (*p* = 0.002) after taking aspirin.

**Discussion:**

Inflammation could be an essential factor in the pathogenesis of subcortical SBIs, and aspirin affects several inflammation-related biomarkers.

## Introduction

Subcortical silent brain infarcts (SBIs) are unrecognized cerebral infarcts in the brain subcortex that do not produce acute stroke symptoms, but are visible as focal lesions on brain computed tomography (CT) and magnetic resonance imaging (MRI) (Vermeer et al., 2007; Norrving, 2015). The annual incidence of SBI is 2 to 4%, and the prevalence ranges from 5 to 62% in population-based cohorts (Fanning et al., 2014b). Patients with subcortical SBIs have a higher risk of symptomatic stroke, depression, and dementia, and a steeper decline in cognitive function than those without such lesions (Fanning et al., 2014a). Thus, detecting subcortical SBIs to prevent secondary deteriorative events, such as symptomatic ischemic stroke and vascular dementia, has generated broad interest.

Inflammation plays a crucial role in the pathogenesis of acute symptomatic infarcts and subcortical SBIs (Wang et al., 2007). Chronic inflammation is linked to the development of atherosclerosis, which is mainly associated with plaque progression and instability in the large arteries (Jayaraj et al., 2019). One of the critical issues in the pathogenesis of SBIs is the underlying inflammatory process and its connection to specific biomarkers.

Aspirin is a well-known medication used for the prevention of secondary stroke and primary prevention in patients with comorbidities (Hart et al., 2000). Besides the anti-thrombotic effect caused by the inhibition of cyclooxygenase-1 (COX-1), which results in thromboxane synthetase, anti-inflammatory effects via the acetylation of COX-2 by aspirin reduce vascular inflammation and stabilize atherosclerotic plaques (Cyrus et al., 2002; Chiang et al., 2004). Inflammatory cytokines have been linked to the severity of chronic stable angina, and aspirin reduces these cytokines (Ikonomidis et al., 1999). Researchers suggest that aspirin also exerts a protective effect against inflammation by modulating cytokines in cerebral ischemia. However, there are no data on how aspirin affects the biomarkers associated with angiogenesis and inflammation in patients with SBI.

To demonstrate the profile of biomarkers related to inflammation in subcortical SBIs and the effect of aspirin on these factors, we selected plasma levels of migration inhibitory factor (MIF) and matrix metalloproteinase-9 (MMP-9) for analysis because MIF and MMP-9 are well-known plasma biomarkers related to inflammatory processes that disrupt blood–brain barrier permeability in acute ischemic stroke (Vandooren et al., 2014; Liu et al., 2018). We also examined plasma visfatin, a proinflammatory cytokine involved in the formation of atherosclerosis, in patients with subcortical SBIs and healthy controls (Lu et al., 2009). Biomarkers were retested after 3 months of aspirin administration to determine the effect of aspirin on the inflammatory cascade in patients with subcortical SBIs.

## Methods

### Study participants

This study was designed as a prospective trial. We recruited 225 consecutive patients who underwent brain MRI at an outpatient clinic between January 2012 and July 2012. Patients with a history of stroke-related symptoms, signal abnormalities on brain MRI indicating acute infarction or hemorrhagic stroke, intra-and extracranial atherosclerotic stenosis (*n* = 187), diagnosed with an active stage of inflammatory disease (*n* = 8), and recent medication changes affecting inflammatory biomarkers within the last 3 months (*n* = 4) were excluded. Consequently, we included 26 patients who started taking 100 mg aspirin daily on the day of initial diagnosis. In addition, ten age- and sex-matched healthy subjects, confirmed via brain MRI to have no subcortical SBIs, no history of cerebrovascular accidents, or risk factors affecting vascular conditions, such as diabetes, dyslipidemia, and smoking, were recruited as controls from Hanyang University Guri Hospital. Before entering the trial, informed consent was obtained from each participant or their legal representative. This study was approved by the Institutional Review Board of Hanyang University Guri Hospital (IRB No. 2010-01-081).

### Clinical information

The baseline characteristics and clinical data of the recruited subjects were collected. Demographic information included age, sex, body mass index (BMI), presence of ischemic stroke risk factors (hypertension, diabetes, hyperlipidemia, and smoking), and medication history. We recorded each subject’s height and weight to calculate BMI using the following formula: BMI = weight (kg)/height (m^2^). Hypertension was defined as systolic blood pressure > 140 mmHg, diastolic blood pressure > 90 mmHg, or the use of antihypertensive medication. We classified the presence of dyslipidemia as a previous diagnosis of the disease and concurrent intake of lipid-lowering agents. The presence of diabetes was characterized by taking antidiabetic drugs with a prior diagnosis of the disease or random blood glucose levels higher than 200 mg/dL.

### Image analysis

MRI was performed on all individuals included in this study using a 1.5-T superconducting magnet (CV/I; GE Medical Systems, Seoul, Korea). Neuroradiologists initially interpreted the brain imaging results without being informed of the patient’s clinical data. Subcortical SBI was defined as a focal hyperintensity on T2-weighted images, at least 3.0 mm in size, with hypointensity on T1-weighted images and a hyperintense rim on fluid-attenuated inversion recovery images found in the brain subcortex (Vermeer et al., 2007; Zhu et al., 2011). We also collected the number, location, and size of the subcortical SBIs.

### Enzyme-linked immunosorbent assays for biomarkers

MIF, MMP-9, and visfatin levels were measured using commercially available quantitative sandwich ELISA kits following the manufacturer’s instructions (R&D Systems, Minneapolis, MN, United States). Plasma samples from patients with SBIs were collected at baseline and after 3 months. The plasma of healthy controls was collected only at the baseline. All samples were stored at −80°C.

### Statistical analysis

We used R software and IBM SPSS Statistics for Windows 26.0 (Chicago, IL, United States) for statistical analyses (version 3.6.0.). A *p*-value less than 0.05 was defined as the threshold for significance. Pearson’s chi-square test was used to compare categorical variables between the two groups. The Shapiro–Wilk test was used to determine normal distribution of continuous variables, and data showing normal distribution were subjected to the Bartlett test to assess the homogeneity of variances. We also used the independent two-sample *T*-test, Welch two-sample *T*-test, and Mann–Whitney test to determine differences between the patient group and healthy controls. To evaluate the effect of aspirin on the biomarkers, we used the Wilcoxon signed-rank test.

## Results

The demographic and clinical data of the 26 patients with subcortical SBIs and ten healthy controls are presented in Table 1. There were no significant differences in demographic characteristics (age, sex, and BMI) between the two groups. Among the patients with subcortical SBI, eight (30.8%) had hypertension, four (15.4%) had diabetes, two (7.7%) had dyslipidemia, and one (3.8%) was a current smoker. The mean number of subcortical SBIs per patient was 1.5. The mean diameter of the lesions was 3.7 mm and ranged from 3.0 to 5.0 mm. A single SBI was detected in 17 patients, and nine subjects had two SBIs. The highest number of lesions per patient was three subcortical SBIs, which were present in three patients. All subcortical SBIs were located in the subcortical areas. The most frequent subcortical SBI regions were the basal ganglia (52.6%) and the subcortical areas (44.7%). Only one patient had SBI in the thalamus.

**Table 1:** Demographic and clinical characteristics of patients with subcortical silent brain infarcts and healthy controls.

	Healthy control (*n* = 10)	SBI group (*n* = 26)	*p*-value
Sex, *n* (%)			0.060^a^
Male	2 (20.0)	6 (23.1)	
Female	8 (80.0)	20 (76.9)	
Age, years (Mean ± SD)	57.6 ± 14.3	64.4 ± 6.9	0.176^b^
BMI, kg/m^2^ (Mean ± SD)	24.6 ± 3.1	24.2 ± 3.6	0.783^b^
Hypertension, *n* (%)	0 (0)	8 (30.8)	
Diabetes, *n* (%)	0 (0)	4 (15.4)	
Dyslipidemia, *n* (%)	0 (0)	2 (7.7)	
Smoking, *n* (%)	0 (0)	1 (3.8)	
Alcohol, *n* (%)	0 (0)	2 (7.7)	
SBI number	–	1.5 ± 0.7	
SBI size, mm	–	3.7 ± 0.8	

a Pearson’s chi-squared test for the sex variable between the healthy control group and the SBI group.

b Welch’s two-sample *t*-test was used for the age variable and an independent two-sample *t*-test was used for the BMI variable between the healthy control and SBI groups after checking normality with the Shapiro–Wilk test and homogeneity of variances by the Bartlett test.

SBI, subcortical silent brain infarcts; SD, standard deviation; BMI, body mass index.

Table 2 and figure present the biomarker levels in patients with subcortical SBIs and healthy controls. In the healthy control subjects, the median MIF level was 58.83 ng/mL (interquartile range (IQR), 44.82–78.97 ng/mL). The median MIF level in the SBI group was 76.75 ng/mL (IQR, 75.19–85.95 ng/mL), presenting a significant difference (*p* = 0.031). MMP-9 levels were also elevated in patients with SBIs compared to healthy subjects (28.25 ± 7.80 ng/mL vs. 38.97 ± 13.68 ng/mL, *p* = 0.026). However, visfatin levels did not differ significantly between the two groups were not significantly different.

**Table 2:** Comparison of biomarker results in the SBI patient group before and after treatment, and the healthy control group.

	Healthy control (*n* = 10)	SBI group before treatment (*n* = 26)	SBI group after treatment (*n* = 26)	*p*-value	
				Healthy vs. SBI before treatment	SBI before treatment vs. after treatment
MIF, ng/mL Median (IQR)	58.83 (44.82–78.97)	76.75 (75.19–85.95)	74.73 (66.70–82.36)	**0.031**	0.280
MMP-9, ng/mL Mean ± SD	28.05 (21.38–34.7)	35.65 (30.63–44.0)	47.4 (32.33–61.05)	**0.026**	0.266
Visfatin, ng/mL Median (IQR)	1.03 (0.90–1.29)	1.00 (0.86–1.16)	0.84 (0.77–0.91)	0.537	**0.002**

IQR, interquartile range; SBI, subcortical silent brain infarcts; MIF, macrophage migration inhibitory factor; MMP-9, matrix metalloproteinase-9; SD, standard deviation.

Bold values indicate *p*-values less than 0.05.

**Figure 1:**
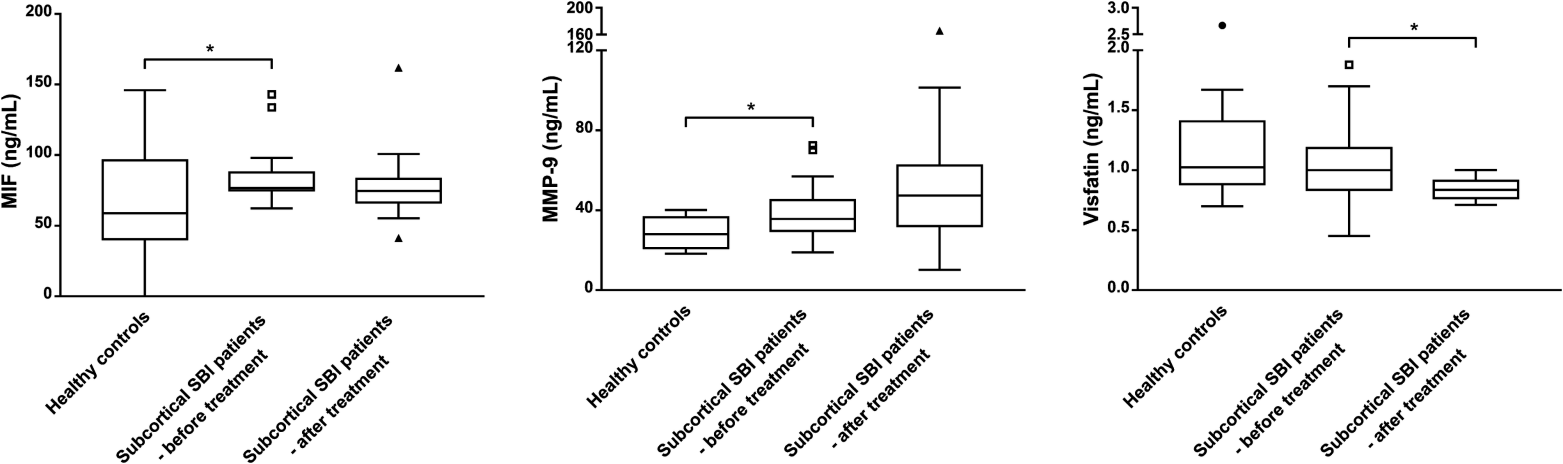
Biomarker levels measured after treatment with aspirin for 3 months in patients with subcortical silent brain infarcts (SBI) compared to levels in healthy controls. MIF and MMP-9 levels, which showed significant differences between healthy controls and the baseline concentrations of patients with SBIs, were not significantly different after treatment with aspirin. Comparisons were made against the control (* *p* < 0.05; Mann–Whitney test). Visfatin levels in patients with SBIs were significantly lower after taking aspirin for 3 months. Boxes indicate 25 and 75% percentiles, with the horizontal median line inside the boxes. Error bars represent 5 and 95% ranges, providing a clear view of the central variability in the data while reducing the impact of extreme outliers. Data points represented as triangles and circles indicate values that lie outside the error bar ranges. The conditions before and after taking aspirin were compared (* *p* < 0.05; Wilcoxon signed rank test).

**Figure 2:**
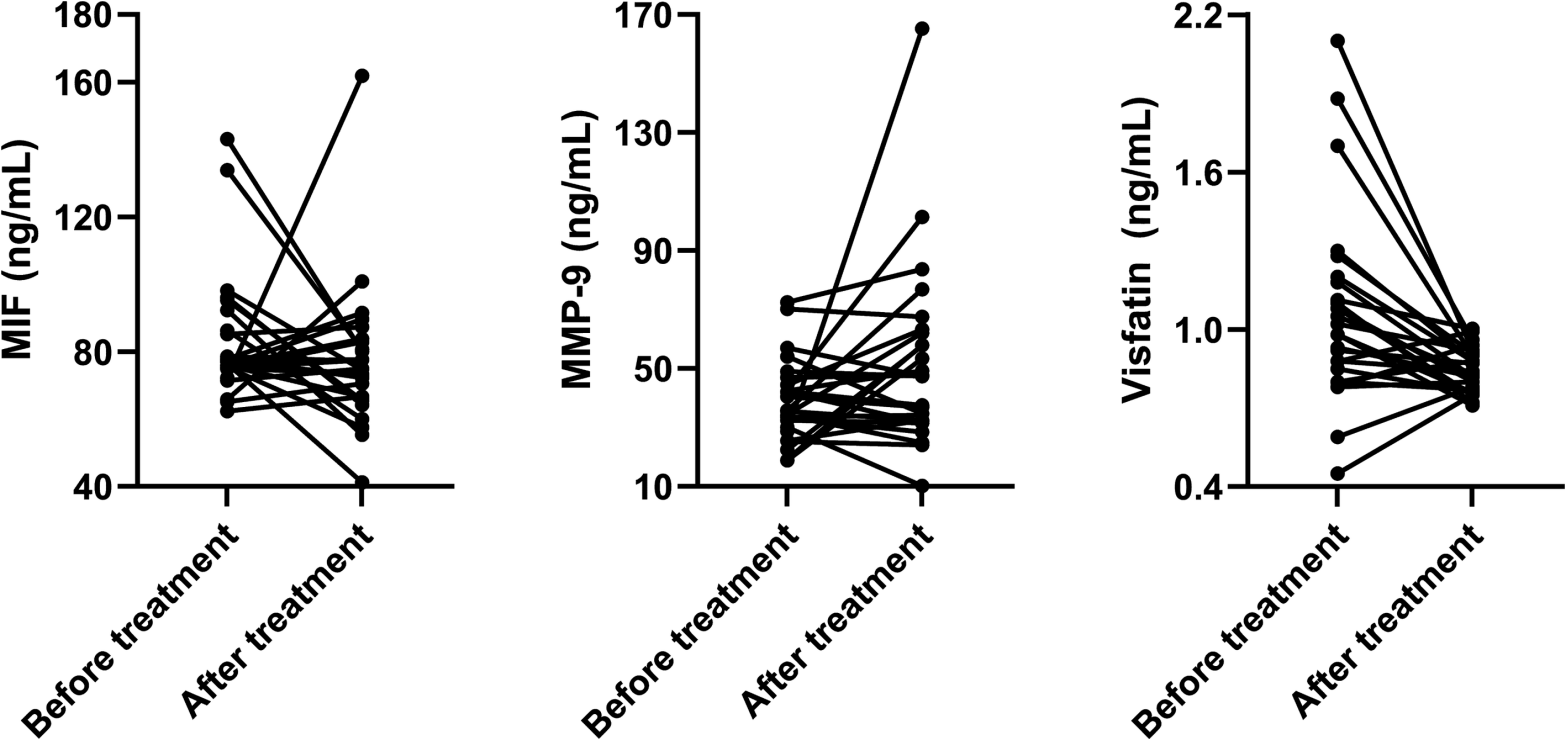
Paired line graphs showing individual changes in biomarkers before and after aspirin treatment in patients with subcortical silent brain infarcts.

Next, we compared biomarker levels at baseline and after taking aspirin for 3 months in patients with subcortical SBIs (Table 2; Figures 1, 2). The baseline median plasma visfatin level in the subcortical SBI group was 1.00 ng/mL (IQR, 0.86–1.16 ng/mL), while the median visfatin level decreased to 0.84 ng/mL (IQR, 0.77–0.91 ng/mL) after 3 months of aspirin treatment (*p* = 0.002). Other biomarkers did not show any significant differences.

## Discussion

This study investigated differences in plasma inflammatory biomarkers between patients with subcortical SBIs and healthy subjects. Higher plasma concentrations of MIF and MMP-9 were detected in patients with SBI than in healthy controls. We also determined alterations in biomarkers after 3 months of aspirin administration. A decrease in plasma visfatin levels was observed, which could be attributed to the effect of aspirin on inflammatory biomarkers.

Various inflammation-related biomarkers have been implicated in the pathophysiology of ischemic stroke. For example, MIF, a neuromodulator, particularly in neurons, promotes neuronal death and aggravates neurological deficits in an experimental stroke model (Inácio et al., 2011). Visfatin is a novel adipokine that is upregulated in ischemic stroke and promotes the expression of inflammatory cytokines and atherosclerosis through vascular smooth cell maturation (Lu et al., 2009). The neuroinflammatory response of MMP-9 in ischemic stroke has also been investigated. Elevated MMP-9 levels result in neurological deterioration in acute lacunar stroke and parenchymal hematoma formation after thrombolytic therapy (Kim et al., 2006; Castellanos et al., 2007). However, MMP-9 also participates in plasticity and recovery throughout the late phase of cerebral ischemia (Wang et al., 2007).

Previous studies evaluated the relationship between inflammatory biomarkers and SBI. Many investigators regard subcortical SBI as sharing a core pathophysiological process with acute symptomatic ischemic stroke. For instance, Hoshi et al. reported that the levels of inflammatory biomarkers, such as high-sensitivity C-reactive protein and interleukin-6, were higher in patients with SBIs than in the healthy control group (Hoshi et al., 2005). We believe that this study strengthens the evidence that subcortical SBIs undergo hypoxic events similar to acute symptomatic cerebral ischemia by presenting higher MIF levels in patients with subcortical SBIs than in healthy controls. Although Sarchielli et al. reported that MIF levels did not differ between groups diagnosed with SBIs and healthy populations (Sarchielli et al., 2013), several additional studies have suggested that MIF expression is upregulated in ischemic conditions (Li et al., 2017; Neumaier et al., 2023). We believe that hypoxic events may cause higher MIF levels during the occurrence of subcortical SBIs. We also found that plasma MMP-9 levels were higher in patients with subcortical SBIs than in healthy subjects. MMP-9 does not exist in the central nervous system but can be detected after cerebral ischemia and breakdown of the blood–brain barrier (BBB) (Rosell et al., 2006), eventually leading to neuronal death (Rosell et al., 2006; Koh et al., 2011). We suggest that elevated MMP-9 levels in patients with subcortical SBIs contribute to the evidence that the BBB is also disrupted in subcortical SBIs, resulting in neuronal injury.

The present study also demonstrated a decrease in plasma visfatin levels after aspirin treatment. Previous studies have reported that higher visfatin levels correlate with disease severity in ischemic stroke and are independent risk factors for atherosclerosis formation (Yin et al., 2013; Kong et al., 2014). Visfatin acts as a proinflammatory cytokine and provokes chronic inflammation by stimulating the maturation of pre-B cells and the expression of inflammatory cytokines in epithelial cells and prolonging neutrophil survival (Lu et al., 2009). Aspirin may block this inflammatory cascade. By acetylating COX-2, aspirin can inhibit COX-2-mediated cell activation and proliferation, and limit the release of these cytokines into the bloodstream (Chiang et al., 2004). We can assume that aspirin’s action on visfatin is important in cerebral ischemia, including SBI, along with disturbing platelet aggregation, oxidative stress, endothelial activation/dysfunction, and other anti-inflammatory reactions (Khan and Mehta, 2005).

While the baseline plasma MIF and MMP-9 levels in patients with subcortical SBIs were significantly higher than those in healthy controls, the plasma MIF and MMP-9 levels did not show significant differences in the patient group after aspirin treatment. Although not statistically significant, plasma MIF levels decreased slightly following aspirin administration. Aspirin is well-known for its acetylation of cyclooxygenases, and previous studies have reported that it may also enhance MIF acetylation in animal models with ischemic injury (Cyrus et al., 2002; Hu et al., 2022). Based on these findings and the results of this study, we can hypothesize that aspirin may not induce a significant absolute reduction in MIF levels in SBIs but could modestly decrease MIF levels through acetylation. Further extensive studies in humans are warranted to validate this hypothesis. Regarding MMP-9, an increasing trend was observed following aspirin treatment, albeit without statistical significance. This result contrasts with prior studies reporting a reduction in MMP-9 levels after aspirin administration in *in vitro* models for atherosclerosis studies (Hua et al., 2009). Since MMP-9 has been implicated in plasticity and recovery during the later phases of stroke, it is plausible that the interaction between MMP-9 and aspirin in asymptomatic SBI may have distinct characteristics (Wang et al., 2007). Further research is needed to explore this interaction in in a more comprehensive manner.

This study had some limitations. First, data were collected from a single center. As a result, we only observed inflammatory biomarkers from the relatively limited sample sizes of patients and control subjects. Second, the diagnostic evaluations of other comorbidities, such as heart and other vascular diseases, which can be risk factors for SBI, were not performed. Third, we cannot explain how aspirin affects inflammatory biomarkers since this was an observational study using the plasma of patients and healthy volunteers. Investigating the molecular details of the mode of action of aspirin to obtain more definite conclusions would strengthen the findings of this study. Lastly, the relatively small sample size may limit the generalizability of the results. Future studies with larger, multicenter cohorts are essential to validate the findings of this study before broader implementation.

Despite these limitations, the present investigation revealed that inflammatory biomarkers were increased in patients with SBIs, suggesting the existence of an inflammatory process in the pathogenesis of subcortical SBI. Furthermore, our study demonstrated downregulation of inflammatory biomarkers associated with inflammation after aspirin administration. Our results also indicate that the anti-inflammatory nature of aspirin regulates inflammatory biomarker profiles in patients with subcortical SBIs. Therefore, we suggest aspirin intake in patients with comorbidities and a higher risk of symptomatic ischemic stroke.

## Data Availability

The raw data supporting the conclusions of this article will be made available by the authors, without undue reservation.
